# The effect of three different techniques used to improve vein prominence on the first peripheral venous catheterization experience in nursing students: a randomized controlled study

**DOI:** 10.1186/s12909-025-07350-0

**Published:** 2025-05-22

**Authors:** Hülya Yılmaz, Dilek Yılmaz, Hava Gökdere Çinar

**Affiliations:** 1 Department of Fundamentals of Nursing, Faculty of Health Sciences, Bursa Uludag University, Bursa, Türkiye; 2 Department of Management in Nursing, Faculty of Health Sciences, Bursa Uludag University, Bursa, Türkiye; 3 Present address: Department of Fundamentals of Nursing, Faculty of Health Sciences, Bursa Uludag University, Gorukle, Bursa, 16059 Türkiye

**Keywords:** Isometric exercises, Near-infrared, Nursing education, Peripheral venous catheterization

## Abstract

**Background:**

Peripheral venous catheterization (PVC) insertion is a complex sensory-motor skill that can be difficult for nursing students to acquire.

**Objectives:**

The authors aimed to examine the effect of three techniques used to improve vein prominence on achievement, knowledge, and psychomotor skills in the first PVC experience in nursing students.

**Methods:**

A single-center, parallel-group, randomized control study. Near-infrared light visualization (*n* = 49) and isometric exercise (*n* = 50) techniques were used in the intervention group, while the standard technique was used in the control group (*n* = 48). Data was collected using Prominence of the Veins, PVC Knowledge Evaluation Form, and Checklist for PVC.

**Results:**

In intra-group comparisons, participants in all three groups showed statistically significant improvements in their pre-and post-test knowledge scores. Regarding PVC psychomotor skills, no differences were seen between the groups in this study. A statistically significant difference was found between the groups in their success rate in acquiring the skill of PVC at the first attempt. It was seen that the most successful group was the infrared light visualization group.

**Conclusions:**

This study highlighted the significant effect of isometric exercise and NIR vascular imaging in increasing vein prominence in nursing PVC training. The findings support the importance of using NIR light visualization technology and isometric exercise training tools for successful PVC placement.

**Registration:**

The trial was retrospectively registered at the ClinicalTrials.gov Protocol Registration and Results System (PRS) (ClinicalTrials.gov ID: NCT06056531).

## Introduction

Peripheral venous catheterization (PVC) is the procedure of inserting a catheter through a patient’s skin into the lumen of a vein, and it is one of the most common skills used in the nursing profession [1]. It is a vital practical skill used by doctors, paramedics, technicians, and registered nurses that involves psychomotor aspects, theoretical and practical knowledge, and manipulation skills [2]. However, the assessment, maintenance, and removal of PVC is primarily the responsibility of nurses [3]. Successful intervention rates vary depending on whether this intervention is specific to the patient (age, ethnicity, dark skin, body structure, adipose tissue accumulation, fluid status, blood velocity, and vessel diameter, obesity) or the underlying clinical picture (anamnesis of chronic disease, problems associated with the circulatory system-peripheral edema, hypothermia, dehydration, septic shock, vasoconstriction, presence of thrombophlebitis, hematoma, ecchymosis due to previous interventions) or the nurse’s experience [4].

PVC insertion is a skill that must be acquired in nursing education. Inadequate education or inaccurate placement of the PVC can increase the risk of complications. One of the recommendations to reduce PVC insertion failure and its complications is to increase nurses’ and nursing students’ education [5]. PVC placement is an invasive intervention challenging to learn and perform. Nursing students often need multiple practice experiences to gain proficiency, and the process frequently leads to anxiety, stress, and fear of failure [6]. PVC skill learning is carried out in two settings, i.e., simulation and clinical implementation [7, 8]. Invasive nursing skills are learned and practiced in preparation for clinical learning experiences with partial-task trainers and peers [9, 10].

The most key point to be taught to students in PVC skills training is techniques to increase vein prominence and visibility in parallel with selecting the correct site and vein [11]. Vein prominence is assessed by methods of inspection and palpation [12]. It was found that considering the difficulty of developing palpation for the first time in PVC training, the lack of hand-eye coordination, and the lack of success resulting in frustration because of critical or non-critical errors, students need more practice with different teaching instruments under the supervision of instructors [13]. For nursing students to develop the manipulation skills required to meet the appropriate clinical learning objectives, various techniques can be used.

A rich vascular anatomy and physiology knowledge is required for ensuring successful PVC learning [14]. During vessel selection by inspection, arteries, and nerves should be identified based on anatomical knowledge of the site (e.g., median nerve). The normal and abnormal characteristics of vessels, such as shape, size, path, patency, and flow, should be examined by palpation technique [15]. Therefore, it is important for nursing students to acquire theoretical and practical knowledge and the necessary skills in accordance with current guidelines [16]. It is also very important to assess whether nursing students have achieved a sufficient level of knowledge in PVC skills to document the learning outcomes in accordance with the Bologna Declaration (a), to support PVC-related patient safety in clinical rotations (b), and to prevent complications (c). In this context, nurse educators should evaluate current educational strategies, and examine current technological equipment and devices, and methods to increase PVC skill learning of students [17].

Near-infrared (NIR) vascular imaging makes veins more visible by taking advantage of the light-reflecting feature of haemoglobin. It creates a dark contrast with skin tissue due to light absorption, and veins are visualized in phosphorescent colour [18]. NIR light visualization and isometric exercise are known to be used in the clinical field to enhance the visibility of veins [19].

## Background

The traditional methods used to increase vein prominence include milking the vein, slapping the skin, applying the tourniquet, negative pressure, warming the area, applying vasodilatation pastes, etc [6]. In addition to the traditional methods of increasing venous prominence, there are modern methods such as multi-spectral cameras [20], robotics systems [21], visible light transilluminators [22], ultrasound [23], NIR spectroscopy [19].

There is a wide variety of clinical trials on NIR. Most clinical trials have focused on PVC in preterm infants and children [19, 24]. In addition, there are studies in which the effect of NIR on PVC skill has been examined in patients diagnosed with hemophilia with a history of difficult vascular access [25], in adult patients admitted to the emergency department [26], in patients diagnosed with rheumatism [27], in patients receiving chemotherapy [28]. All these study results support the inclusion of NIR vascular imaging in clinical practice, highlighting its potential benefits for patient safety. While no medical technology is entirely without risk, the evidence suggests that NIR is a safe and effective tool. Fukuroku et al., in a study comparing the tourniquet and NIR methods, indicated that NIR was effective for nursing students in locating difficult-to-detect veins [29].

There is a scarcity of clinical trials on isometric exercises. A study examining the effects of handgrip exercise training with and without venous restriction on muscle strength and vascular responses noted that it could potentially increase vein prominence [30]. Rus et al., assessed the impact of daily handgrip training combined with intermittent compression on the forearm veins of chronic haemodialysis patients. Results showed significant increases in forearm circumference and venous diameter after eight weeks of training, indicating that handgrip exercises can enhance vein prominence through increased blood flow and vascular adaptation [31]. Another study involving healthy individuals found that fist clenching significantly increased the visibility of both dorsal metacarpal and cephalic veins. Depending on the specific vein assessed, participants improved after an average of approximately 10 to 22 fist clenches [28].

Among the advantages of NIR light is that the vein anatomy is imaged as the procedure occurs. This unique advantage can allow students to see the 2-dimensional anatomy and the potential complications that may arise. For this purpose, this study evaluated the effects of three techniques used to improve vein conspicuity on nursing students’ success, knowledge, and psychomotor skills during their first peripheral venous catheterization experience. Three techniques used to increase vein prominence were: vein visualization with NIR light, vein dilation through isometric exercise, and the traditional tourniquet method, which served as the control. This study is the first finding in nursing education that examines practical skill-learning techniques for increasing venous prominence fullness after isometric exercise with a stress ball and vascular imaging under bright light with NIR. The research questions were as follows:

1) Is there a difference in initial PVC success among nursing students during the vein prominence improvement intervention with three different techniques?

2) Is there a difference in initial PVC knowledge and psychomotor skill scores among nursing students during the vein prominence improvement intervention with three different techniques?

## Method

### Design

**Fig. 1:**
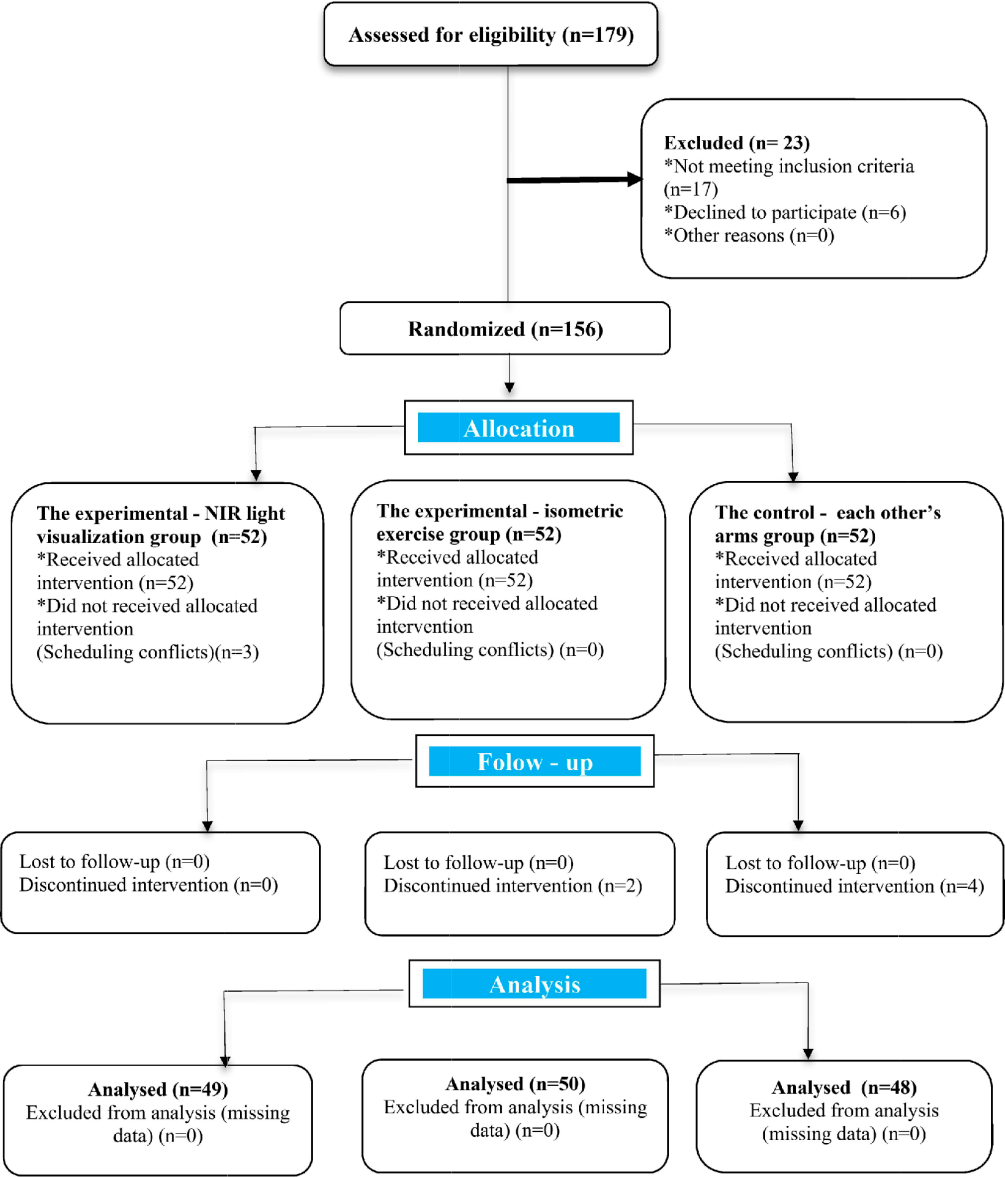
CONSORT flow diagram of the study

This study was performed as a single-centre, parallel-group, randomized controlled study using a pre-test and post-test design. Figure 1 demonstrates the CONSORT flow diagram of this study.

### Participants and data collection

The research was conducted by enrolling first-year nursing students in western Türkiye. The universe of the study consisted of nursing students (*n* = 179) who were attending the Department of Nursing at a university and taking the course module for basic nursing skills between February and July 2022. The inclusion criteria for participants comprised individuals who had not previously undergone training in PVC skills, those who could certify their vaccinations as protective health workers, and those who volunteered to participate. Participants were excluded from the study if they had been diagnosed with a chronic illness, a bleeding disorder, or a psychiatric condition; if they had not received vaccinations for HBV, HCV, or HIV; or if they did not complete both the pre-test and post-test. Students who were excluded or withdrawn from the study continued their standard education.

In the preliminary study conducted to calculate the sample size, power analysis was performed in G*Power Version 3.1.9.6 statistical software. In the success rate comparison between the groups, Cohen stated that when the effect size was 0.3 at the medium level, at least 48 participants should be included in each group at the 80% power and 5% significance level [32]. After the training and the PVC knowledge test, students were divided by the stratified sampling method into three groups of low, medium, and high levels according to their academic grade averages. This selection removes doubts concerning bias in the randomization processes. This, in turn, strengthens the internal validity of the research, allowing for more robust conclusions [33].

An equal number of participants from each level were distributed to a stratified sample group. Then the researchers used number 1 for the NIR light visualization training group, number 2 for the isometric exercise training group, and number 3 for the control group. These numbers were placed in a dark box. Participants selected numbers from the box and were assigned to groups in line with their numbers. This randomization process was repeated until the groups had equal participants. The study was completed with a simple random sampling method, with 147 students participating in the NIR light visualisation (*n* = 49), isometric exercise (*n* = 50), and control (*n* = 48) groups. Data were collected between February and July 2022 in the spring academic semester. Blinding was not implemented in this study, limiting the ability to mitigate bias; however, due to the nature of the intervention and procedure, blinding was deemed impractical and ethically challenging, acknowledging this as a notable limitation of the research.

#### Phase 1: design of educational intervention

**Fig. 2:**
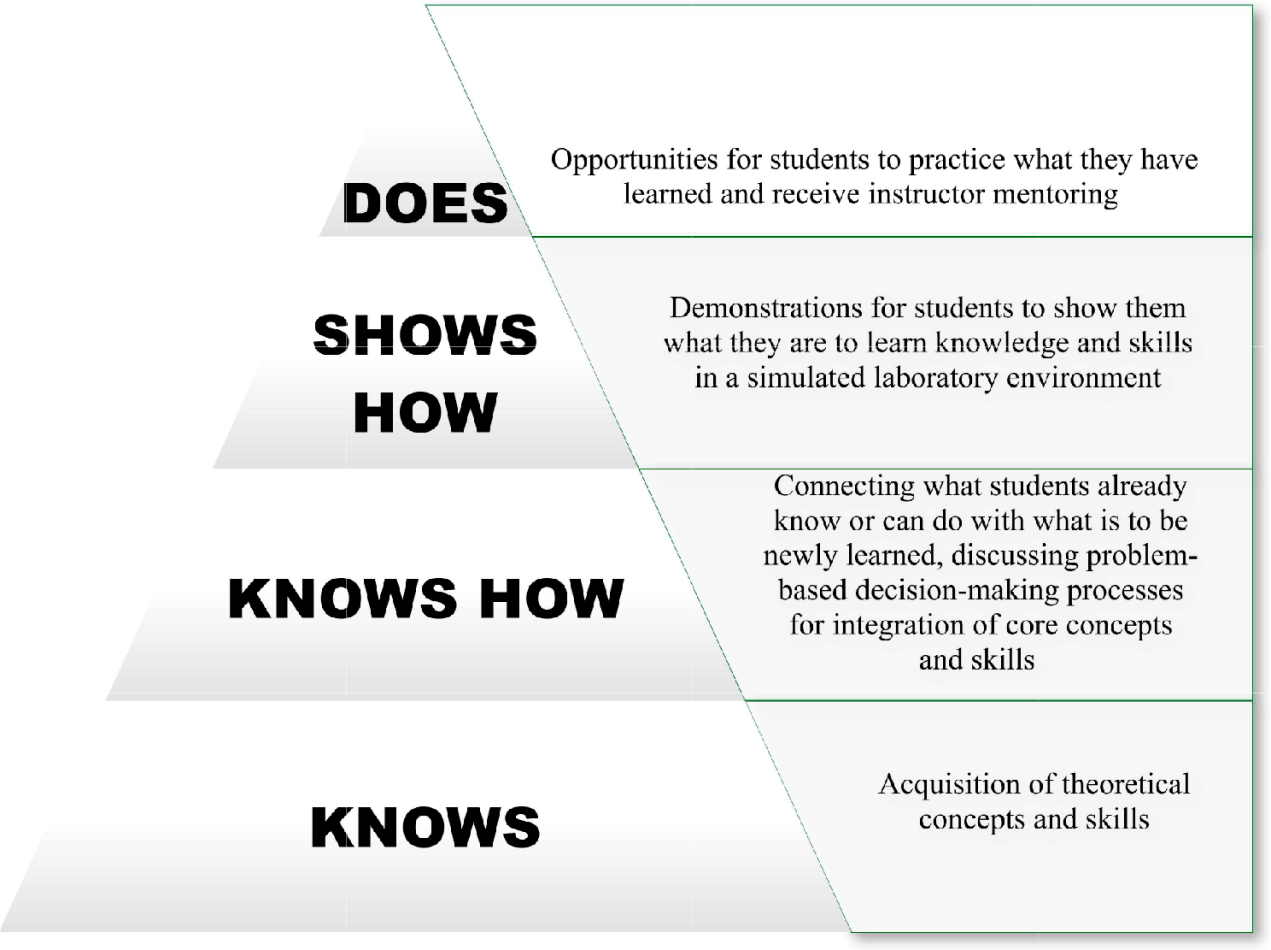
Miller Pyramid

For this study, the educational intervention was designed according to a synthesis of instructional theories proposed by the Miller Pyramid. The steps of this pyramid are as follows: (1) *Knows*: acquisition of theoretical concepts and skills (2) *Knows How*: connecting what students already know or can do with what is to be newly learned, discussing problem-based decision-making processes for integration of core concepts and skills (3) *Shows How*: demonstrations for students to show them what they are to learn knowledge and skills in a simulated laboratory environment (4) *Does*: opportunities for students to practice what they have learned and receive instructor mentoring [34]. A detailed scheme is shown in Fig. 2.

#### Phase 2: theoretical information

Peripheral venous catheterization is included in the practice in the fundamentals of the nursing curriculum. PVC training was explained face-to-face to all participants in two parts. The first part consists of a presentation including the anatomy, physiology, and pathophysiology of peripheral vessels; definition of PVC; steps to be followed in the procedure of PVC placement; internal and external factors affecting the PVC process; techniques that can be used to increase vein prominence; catheter selection; catheter replacement, and complications (60 min). The following topics are mentioned in the second part: catheter care; evaluation of complications related to PVC; maintenance of infusion; calculations of the number of drops per minute; technological infusion pump sets used for IV fluid replacement; flash solutions; anticoagulants; and topical agents (120 min) [35, 36].

#### Phase 3: Pre-test and demonstration with IV arm task trainer

After the theoretical instruction, all participants were administered the pretest utilising the PVC knowledge assessment form. After 10 days, laboratory applications began. PVC skills on a plastic IV arm were demonstrated to all participants by the first author. Then, the participants were requested to practice the skill at least once on a plastic IV arm under the supervision of the researchers. The participants were allowed to repeat the skill without a time limit until they felt competent in the PVC placement, and meanwhile, their questions were answered.

#### Phase 4: intervention

After the demonstration stage, the participants experienced placing PVC on each other under the supervision of the researchers. At this stage, everyone completed the PVC procedure according to their group. Each participant was allowed to practice the skill two times at most. In order to prevent the communication of the participants with each other, three separate simulation rooms were used in the Skills Laboratory.

### Data collection

#### Performing the application on the NIR light visualisation group

Accuvein^®^ is not user-dependent, unlike ultrasonography, which depends on user skill. Before or during the taking of a blood sample, it is possible to quickly and broadly evaluate the path and diameter of subcutaneous veins by using this equipment.

A participant practicing the skill performed a PVC insertion in another participant’s arm veins with NIR light visualization. The authors held the device at an equal distance of 20 cm. Two independent researchers assessed the psychomotor skills of the participants using a checklist. The researchers used a standard chronometer to measure and record the PVC procedure period. The duration for the participants to select the necessary materials was not included. The chronometer was started after the application of the tourniquet. The chronometer was stopped when the steel needle of the cannula was removed. After the application of the tourniquet, the participants were asked about the degree of vein prominence under NIR light, and their subjective evaluations were recorded. The participants were allowed to use the inspection and palpation methods in the grading of vein prominence. Procedure success was recorded. Blood return upon removing the steel needle was considered a success criterion. The PVC procedure was considered a failure if no blood came into the cannula. Regardless of the success of the PVC placement, the catheter was immediately removed, and no liquid medication, etc. was given. A cotton swab was applied to the treated site.

#### Performing the application on the isometric exercise group

The participants in this group were provided with isometric exercise before the skill. The participants were given two stress balls and explained how to use them. The stress balls used in the study were 6 cm diameter, yellow-coloured, medium-hard, and high-quality silicone. The participants were told to squeeze and relax the stress ball in their hands after counting from one to three, to continue squeezing the ball, and to concentrate for 15 min until the process started. After performing the isometric exercise and the application of the tourniquet, the participants were asked about the degree of vein prominence, and their subjective evaluations were recorded. The remaining stages were performed in the same manner in all groups.

#### Control group

Participants in this group performed PVC directly on each other without any intervention. After the application of the tourniquet, the participants were asked about the degree of vein prominence, and their subjective evaluations were recorded. The remaining stages were performed in the same manner in all groups.

#### Phase 5: post‑intervention survey

Two weeks after the completion of the intervention stages, all participants were given a final test through the PVC knowledge evaluation form.

## Instruments

### Prominence of the veins

Vein visibility is evaluated using a five-step scale [36, 37]. Participants are evaluated using this scale: 1 = no veins visible or palpable, 2 = visible but not palpable, 3 = barely visible and palpable veins, 4 = visible and palpable veins, and 5 = clearly visible and easily palpable veins.

### Peripheral venous catheterization knowledge evaluation form (PVC-KEF)

The researchers used this form to define participants’ knowledge levels on the topic of the PVC procedure. This form consisted of 20 questions on PVC topics. The lowest possible score on the form was 0, and the highest was 100. A higher total test score was interpreted as a higher level of PVC knowledge [38]. The relevant literature research was utilised for the arrangement and formation of the questions [11, 35, 36]. This form was submitted to five experts in the nursing field and five experts in measurement evaluation in education for their views. According to the analysis of the expert views, the content validity index score was 0.98. The 20-question form, which was found to be suitable, was later given to five third-year nursing students to assess their comprehension, and their feedback was taken.

### Checklist for peripheral venous catheterization (C-PVC)

The researchers prepared this checklist based on the literature [11, 35]. It covered 20 steps in the PVC procedure. The evaluation indicated that the student had completed (2 points), incompletely performed (1 point), or not performed (0 points) each stage of the procedure. According to the test score, which was between 0 and 40 points, a higher total test score in the assessment demonstrated that the student’s PVC skills level was high. According to the analysis of expert opinions, the content validity index was 1.0. After deciding on the appropriate checklist, the 20 steps were delivered to the instructors excluded from the study (*n* = 5) to evaluate its comprehensibility, and their feedback was received. The psycho-motor stages of participants’ PVC implementation skills taught by different instruction methods were assessed with a skill performance test by two independent researchers. The ICC coefficient was found to be 0.899.

### Ethical considerations

This randomize, controlled using a pre-test and post-test study was approved by the Clinical Research Ethics Committee of redacted University Medicine Faculty (Approval No: 22/09/2021-13/22). The study was registered in the Protocol Registry System at Clinicaltrials.gov (no: NCT06056531) and was conducted in accordance with the principles of the Declaration of Helsinki. Written permissions were obtained from the institutions where the study was performed. Informed consent was obtained in writing from the eligible students using an Informed Consent Form, and they were informed about the procedures and research, such as aims, subjects, and methods. All information gathered from nursing students was kept as private as possible.

### Data analysis

The Shapiro-Wilk test examined whether the data demonstrated a normal distribution. For quantitative data, descriptive statistics were expressed as the mean and standard deviation; for qualitative data, they were defined as the median frequency (min-max) and percentage. If one-way analysis of variance failed to demonstrate normal distribution in more than two group comparisons for normally distributed data, the Kruskal Wallis test was applied. The Wilcoxon sign-rank test was utilised for statistical differences between dependent groups. Categorical data were analysed using the Pearson Chi-square, Fisher-Freeman-Halton, and Fisher’s Exact Chi-square tests. A multiple comparison test, the Bonferroni test, was utilised to determine significance. The agreement between raters was investigated using the intraclass correlation coefficient. The threshold for significance was set at 0.05. The statistical package of IBM SPSS 28.0 was used to analyse the data.

## Results

### Participants’ characteristics

The average ages of the participants incorporated in the research were 19.71 ± 1.36 years in the NIR light visualisation group, 19.22 ± 1.56 years in the isometric exercise group, and 19.66 ± 2.66 years in the control group. Thus, there was no noticeable difference between the groups. (F (2;144) = 0.973; *P* =.381). While 85.0% of the participants were female, 15.0% were male. It was found that 80.3% of the students participating in the research performed PVC on the antecubital fossa vein, 61.9% performed the procedure on the right arm, 99.3% used a 22G cannula, 45.6% were successful at the first attempt in the PVC procedure, and 49.4% were successful at the second attempt (Table 1).

**Table 1:** Peripheral venous catheterization skill’s attempt characteristics (*n* = 147)

		*n* (%)
Selected vein region	antecubital fossa	118 (80.3)
	basilic	7 (4.8)
	cephalic	9 (6.1)
	dorsal metacarpal	8 (5.4)
	accessory	1 (0.7)
	radial	4 (2.7)
Selected arm	right	91 (61.9)
	left	56 (38.1)
Size of cannula	22G	146 (99.3)
	24G	1 (0.7)
Success of the first PVC attempt	successful	67 (45.6)
	unsuccessful	80 (54.4)
Success of the second PVC attempt	successful	39 (48.8)
	unsuccessful	41 (51.2)

### The success rates of the PVC attempts and prominence of the selected vein

Table 2 shows that although the fifth degree of vein prominence was at its highest level in the NIR light visualisation group with 28.6%, there was no statistically significant difference between groups (c2 = 2.188; *P* =.335). There was a statistically significant difference between the groups in the proportion of individuals who successfully mastered the PVC skill at the first attempt (c2 = 15.201; *P* <.001). It was seen that the most successful group was the NIR light visualisation group (67.3%) compared to the isometric exercise group (40%) and the control group (29.2%). Unsuccessful participants (*n* = 80) were allowed to make a second attempt. There was no statistically significant difference between groups regarding PVC skill acquisition at the second attempt (c2 = 0.319; *P* =.852) (Table 2). After an unsuccessful attempt at PVC, the catheter was immediately removed. The pressure was applied to the affected area for five minutes with a 70% alcohol swab containing 2% chlorhexidine. The affected limb was elevated, and assessment and documentation of the appearance of the site and associated signs and symptoms were continued.

**Table 2:** Comparison of the prominence of the selected veins and success rates PVC attempt

		Intervention Group NIR light visualization (*n* = 49)	Intervention Group isometric exercise (*n* = 50)	Control Group (*n* = 48)		
		n (%)	n (%)	n (%)	χ2	*P* value
Prominence of the selected vein	1^st^	1 (2.0)	2 (4.0)	2 (4.2)	2.188	0.335
	2^nd^	6 (12.2)	6 (12.0)	4 (8.3)		
	3^rd^	10 (20.4)	9 (18.0)	18 (37.5)		
	4^th^	18 (36.8)	19 (38.0)	15 (31.2)		
	5^th^	14 (28.6)	14 (28.0)	9 (18.8)		
First PVC attempt	successful	33 (67.3)	20 (40.0)	14 (29.2)	15.201	0.001*
	unsuccessful	16 (32.7)	30 (60.0)	34 (70.8)		
Second PVC attempt	successful	9 (52.9)	13 (44.9)	17 (50.0)	0.319	0.852
	unsuccessful	8 (47.1)	16 (55.1)	17 (50.0)		

NOTE. Pearson chi-square and Fisher-Freeman-Halton tests were used, *p <.05

Prominence of the vein: 1^st^ grade: veins not visible or palpable, 2^nd^ grade: veins visible but not palpable, 3^rd^ grade: veins barely visible and palpable, 4^th^ grade: veins visible and palpable, 5^th^ grade: veins clearly visible and easily palpable

### The distribution scores of instruments according to groups

There was no significant difference was found between the groups in mean duration of vein determination (c2 = 4.064; *P* =.131) or in first PVC attempt duration (c2 = 3.735; *P* =.155) (Table 3). The pretest, post-test, and median score differences of the participants incorporated in the research on the PVC-KEF were summarised. In intra-group comparisons, participants in all three groups showed statistically significant improvements in their pre-and post-test scores (NIR light visualisation: Z = -4.191; *P* =.001, isometric exercise: Z = -2.166; *P* =.03, and control: Z = -2.932; *P* =.003). There were no significant differences in post-test scores between the groups (*P* >.05). Similarly, the mean C-PVC scores did not differ significantly between groups (*P* >.05) (Table 4).

**Table 3:** Comparison of groups the duration of vein determination and PVC attempt

	Intervention Group NIR light visualization (*n* = 49)		Intervention Group isometric exercise (*n* = 50)		Control Group (*n* = 48)			
	Min– Max (Median)	Mean (SD)	Min– Max (Median)	Mean (SD)	Min– Max (Median)	Mean (SD)	χ2	*P* value
Vein determination duration	6– 118 (37)	40.79 (30.21)	2– 318 (27)	46.46 (56.75)	5– 259 (41)	60.58 (58.09)	4.064	0.131
First PVC attempt duration	77– 315 (175)	179.95 (60.86)	68– 455 (149)	169.48 (82.05)	82– 457 (177.5)	193.12 (83.85)	3.735	0.155
Second PVC attempt duration	53– 276 (173)	162.70 (55.32)	70– 444 (164)	176.14 (91.20)	77– 453 (184.5)	207.44 (89.31)	4.112	0.128

NOTE. Kruskal Wallis H Test, Wilcoxon Signed Ranks Test

The time is calculated in seconds

**Table 4:** The distribution scores of instruments according to groups

	Intervention Group NIR light visualization (*n* = 49)		Intervention Group isometric exercise (*n* = 50)		Control Group (*n* = 48)			
	Min– Max (Median)	Mean (SD)	Min– Max (Median)	Mean (SD)	Min– Max (Median)	Mean (SD)	χ2	*P* value
Pretest PVC- KEF	25.00 - 85.00 (70.00)	63.26 (14.80)	25.00 - 85.00 (60.00)	60.90 (13.80)	20.00 - 85.00 (65.00)	62.08 (13.59)	1.175	0.556
Posttest PVC- KEF	30.00 - 90.00 (75.00)	70.61 (14.20)	30.00 - 85.00 (70.00)	65.40 (14.10)	35.00 -100.00 (70.00)	69.27 (15.19)	3.508	0.173
	Z = - 4.191, *p* =.001^a^	Z = - 2.166, *p* =.03^a^	Z = - 2.932, *p* =.003^a^					
C– PVC	22.00 - 40.00 (35.00)	33.73 (4.38)	22.00 - 40.00 (33.50)	32.98 (4.74)	15.00 - 40.00 (34.00)	32.35 (5.49)	1.203	0.548

NOTE. Kruskal Wallis H Test, Wilcoxon Signed Ranks Test, ^a^*p* < 0.05

Abbreviations: SD, standard deviation; Min, minimum; Max, maximum; PVC- KEF, Peripheral intravenous cannulation knowledge evaluation form; C - PVC, Checklist for peripheral intravenous cannulation

## Discussion

The prominence of peripheral veins is attributed to variables such as the body structure, ethnicity, fat tissue accumulation, blood flow rate, genetic vascular pattern and problems, dehydration and hypovolemia, presence of oedema, age, and obese status of the individual [38]. Due to these reasons, the prominence of veins varies significantly from individual to individual. On examination of the studies on the use of NIR light visualization, increased vein prominence and better vein visibility were reported to be obtained [40, 41]. Chiao et al. detected that the use of NIR light visualization significantly improved vein prominence by 4 times in obese participants and by 3 times in morbidly obese participants, compared to the traditional method. In the same study, dark-skinned African American participants had better vein prominence than the control group [40]. Like the literature, the results of the current research report an increase in the vein prominence in the NIR light visualization training group (grade 4) by 36.8%, while the isometric exercise training group (grade 4) has the highest rate of 38%. Current research results, which state the effectiveness of NIR in finding difficult-to-detect veins for nursing students, similar to Fukuroku et al., indicate that vein prominence has improved in the groups of NIR and isometric exercise [29]. It was reported that the use of NIR light visualization could visualize the hand veins of all babies and children; however, the veins in the antecubital fossa could not be visualized in 4 patients [42]. In another study, one out of three operators reported that the use of NIR light visualization prevented venipuncture while performing PVC [26]. In another study, dissatisfaction with the vein visibility of NIR light visualization was stated as NIR light caused the veins to appear larger than they were, and the venous depth is not fully understood [43]. These differences in NIR light visualization evaluations may result from constantly evolving device versions. In another study, the effect of palm opening and closing, NIR light visualization, and standard methods on vein prominence was examined. The degree of vein prominence was 35.6% in both the NIR light visualization and standard teaching groups, compared to 11.11% in the isometric exercise group [28]. The difference between the results may be due to the difference in the patient population, in that participants received chemotherapy in the study of Eren and Çalışkan, while the vein prominence was graded in healthy adults in the present study.

The success of PVC in healthy adults depends on the visibility and palpability of the veins and the skill, experience, and skill of the operator [44]. The success of NIR light imaging used in clinical trials for PVC placement remains controversial in the paediatric population. Compared with the standard NIR light visualization method, results were obtained such that PVC reduced the number and duration of the intervention and facilitated the procedure [40] Similarly, based on the results of the current study, the success rate of PVC placement on the first or second attempt was higher in the NIR light visualization group compared to the other groups. Contrary to these results, some studies indicated that the use of NIR light in the paediatric population did not reduce the number or duration of PVC interventions, and did not provide any advantage over the standard method [19, 24]. In one clinical study, there was a small increase in forearm flow velocity with 20 min of isometric palm squeezing exercise five days a week, but the results were not significant. Therefore, the effect of hand grip exercises in increasing the PVC success rate in the absence of peripheral complications was not supported [45]. When examining the findings of the current study, the groups with the highest success rate in the first attempt were NIR light visualization, isometric exercise, and control (respectively, 67.3%, 40.0%, 29.2%). In the present study, participants in the isometric exercise group had a lower success rate than those in the NIR light visualization group but a higher rate than the control group. These differences may be attributed to biochemical and physical variations between healthy participants (nursing students) and patient populations (e.g., chemotherapy patients). Although the isometric exercise group had the shortest first PVC attempt duration, no statistically significant difference was found between the groups. Similar to the results of the current study, in another study conducted in a paediatric blood collection centre, the use of NIR light was detected not to differ from the standard method in terms of the success and period of blood collection at the first attempt [46]. According to the authors’ experience, the use of NIR light may have caused a loss of time as it aroused students’ curiosity but was also a distracting activity element.

Theoretical knowledge of PVC is essential for successful cannulation. In a randomized controlled study conducted with nurses working in the departments of internal medicine and surgery, it was determined that a training program using a computer-based simulator increased the level of success and knowledge in PVC application significantly [13]. In another study, it was stated that PVC training in a mixed reality simulator increased the knowledge level of students [47]. Similar to the results of this study, on evaluation of the PVC knowledge levels of the participants in the present study, the knowledge level of the final test was detected to increase compared to the knowledge level of the pre-test in all three groups. In the present study, it was determined that the pre-test knowledge scores of the participants in the isometric exercise group were lower than the other groups, and the final test scores increased more than the other groups. There are also studies indicating that the training in the virtual simulator is not effective in increasing the knowledge level of the students [48]. The reason for this difference may result from the fact that all participants were given detailed theoretical information in the present study, all participants practiced the PVC skill steps in the laboratory as much as they wanted, and in addition, they tried the PVC intervention on each other in reality. The techniques used in this research can improve students’ understanding and PVC knowledge in the future. Further investigation into the factors influencing the lack of significant differences in PVC knowledge levels and skill performance among the groups is warranted. Potential considerations may include variations in individual learning styles, prior experience with venipuncture techniques, or the effectiveness of instructional methods. Exploring these aspects could provide valuable insights for refining training approaches and optimizing skill acquisition in nursing education and clinical practice.

Learning clinical psychomotor skills is an integral part of nursing education [49]. In the present study, no difference was found between groups in terms of PVC psychomotor skills. However, examining the other studies, NIR light visualization was detected to increase the level of PVC psychomotor skills [50]. In the literature review, similar studies are seen, which compare the standard training and the simulation method in the development of PVC skills. Some studies reported higher PVC skill performance scores in participants receiving simulation training than those receiving traditional training [10, 51]. This difference may be due to differences in the duration and content of training, measurement methods, and evaluation criteria used in other studies.

### Limitations

The study can only be generalized to a certain extent because it was conducted in only one facility. Even though all the instruments underwent preliminary content validity testing for this study, more testing in a broader range of contexts and populations is required to improve their applicability. These results cannot be generalized to clinical PVC attempts regarding success rates or results. Because of the nature of the device and the venipuncture procedure, blinding could not be performed. The study was conducted over a limited timeframe, outcomes were focused on immediate or short-term success.

## Conclusion

This study highlighted the significant effect of isometric exercise and NIR vascular imaging in increasing vein prominence in nursing PVC training. The findings support the importance of using NIR light visualization technology and isometric exercise training tools for successful PVC placement. On evaluating the PVC knowledge levels of the participants in the study, the knowledge level of the final test was detected to increase compared to the knowledge level of the pre-test in all groups. Regarding PVC psychomotor skills, no differences were seen between the groups in this study. Strategies must be evaluated to enhance the PVC insertion technique before graduation. It can be recommended that different teaching techniques be continued under instructor supervision for students in the procedure of PVC placement.

## Data Availability

The datasets generated and analyzed during the current study are not publicly available due [to privacy and ethical restrictions] but are available from the corresponding author upon reasonable request.

## Abbreviations

PVC: Peripheral venous catheterization
NIR: Near-infrared
KEF: Knowledge Evaluation Form
C: PVC-Checklist for peripheral venous catheterization
